# The horror of wrong-site surgery continues: report of two cases in a regional trauma centre in Nigeria

**DOI:** 10.1186/s13037-014-0053-2

**Published:** 2015-01-31

**Authors:** Arinze Nwosu

**Affiliations:** National Orthopaedic Hospital, Enugu, Nigeria

**Keywords:** Wrong- site surgery, Medical errors, Patient safety, “Universal protocol”, “WHO surgical safety checklist”

## Abstract

**Background:**

Wrong- site surgeries are iatrogenic errors encountered in the course of surgical patient management. Despite the ‘never do harm’ pledge in the ‘Hippocratic Oath’ drafted in 5^th^ century BC, man is after all human, with this limitation manifesting in the physician’s art despite his best intention. Beyond the catastrophic consequences of wrong- site surgery on the patient and surgeon, and the opprobrium on the art of medicine, the incidents have come to be regarded as a quality-of-care indicator. Orthopaedic surgery is a specialty with a preponderance of this phenomenon and the attendant medico-legal issues relating to malpractice claims. Consequently the specialty had pioneered institutional initiatives at preventing these ‘friendly-fires’. Awareness and implementation of these initiatives however remain low in many parts of the world, hampered by a culture of denial and shame.

**Case presentation:**

This report presents two cases of wrong-site surgery following trauma from road-traffic accident. The first case was a closed reduction of the ‘wrong’ dislocated hip in the trauma/emergency unit under the care of senior residents, while the second case was attempted wrong-site surgery on the right leg in a patient with fracture of the left tibia, in conjunction with bilateral femoral fracture and right radio-ulnar fracture; by an experienced Chief Consultant Orthopaedic Surgeon operating elective list. Both are orthopaedic cases, each with some trauma to both lower extremeties. Neither of the cases was formally mentioned anywhere in clinical discourse in the hospital, much less a formal report or audit.

**Conclusion:**

There was no formal, institutionalized process to prevent wrong-site surgery in the health institution and this could have been largely responsible for these incidents. An open, mandatory process of reporting such incidents for relevant audit and awareness is necessary, as a mechanism for prevention rather than blame or punishment.

## Introduction

Medical errors are a common cause of mortality and morbidity among patients. Wrong- site surgery refers to the constellation of medical errors involving surgical procedures on the wrong site of the body, wrong person or wrong procedure.

Wrong- site surgery considered a rare and random event was hitherto muted, unpublicised and ignored in professional discourse. The National Patient Safety Agency of the United Kingdom had classified wrong-site surgery as “Never Events”; serious, largely preventable patient safety incidents that should not occur if the available preventive measures were implemented whereas The Joint Commission on Accreditation of Healthcare Organizations of the USA (JCAHO) classifies it as “Sentinel Event”; an unexpected occurrence in a healthcare setting resulting in serious physical or psychological injury, or risk thereof to a patient, not related to the natural course of the patients illness and being the most frequent sentinel event accounting for 13.4% of such events reviewed by the Joint Commission between 1995–2010. Thus wrong-site surgery is considered indicative of serious underlying patient-safety problem.

Despite a long history of these never-events, a formal expanded process to address it only got underway in 1999 with the establishment of the JCAHO. Prior to this time however professional medical bodies had recognised the importance of medical errors and commenced the implementation of patient-safety programs with pioneering effort of the American Society Of Anesthesiologists; “Anesthesia Patient safety Foundation” in 1985, Canadian Orthopedic Association “Operate Through Your Initials” in 1994 and the American Academy Of Orthopedic Surgeons “Sign Your Site” in 1997. The Joint Commission’s “Universal Protocol” incorporating a pre-procedure verification, site marking and a ‘time-out’ immediately before the procedure has been in use in the USA since 2004. By 2008, the “WHO Surgical Safety Checklist” protocol categorised as ‘Sign-in’ , followed by ‘time-out’ and ‘Sign-out’ which goes beyond prevention of wrong-site surgery to encompass measures that promote safe anaesthesia, teamwork and prevention of surgical site infection was published as a product of the WHO Second Global Patient Safety Challenge: “Safe Surgery Saves Lives”. These interventions, as integral part of patient-safety culture are intended to eliminate wrong-site surgery; eventhough the latter has been known to occur in spite of full compliance with all preventive processes and checks. It is thus pertinent to state that total abolition of wrong- site surgery may be far- fetched in view of the multiplicity and complexity of processes that could result in these errors [1,2]. This stark reality following ten years of implementation of the “Universal Protocol” in the United States propels the need for further measures to address some of the inherent pitfalls [3].

In a study [4] 9,744 paid malpractice settlements for surgical “never events” in the United states from 1990 – 2010 amounted to $1.3b; with mortality occuring in 6.6% of the patients, permanent injury in 32.9%, and temporary injury in 59.2%. Thus the cost of these events to the healthcare system and the enormous harm to the patients call for vigorous attention.

Wrong- site surgery is neither alien, nor rare in Nigeria, despite the culture of denial and conspiratorial concealment. In a cross-sectional questionnaire-based study involving 171 dental surgeons, 13% reported having extracted a wrong tooth; whereas 25% were aware of the Universal Protocol for preventing wrong- site, wrong- procedure and wrong- person surgery out of which only a third have read the protocol [5]. These two cases being presented arguably represent the first actual report of wrong- site surgery cases in Nigeria, despite the significant prevalence of these ‘never events’ in our practice as suggested by the survey carried out by Adeyemo et al. [5]. It is thus obvious that wrong- site surgery is grossly under-reported in Nigeria.

### Case 1

A 75 year old female, retired civil servant, presented on June 8, 2011 with inability to bear weight on the left lower limb following road-traffic accident. She is a known asthmatic and hypertensive patient. The attending doctor in the trauma unit had noted abnormal posture of the left lower limb on examination, but made a provisional diagnosis of fracture/dislocation of the right hip. On review the senior resident in orthopaedics noted external rotation and shortening of right lower limb, but made a diagnosis of fracture/dislocation of the left hip. A radiologic report showed central right hip dislocation, with acetabular fracture. The patient was scheduled for reduction of hip dislocation under anaesthesia and tibial steinmann’s pin insertion. The surgeon’s findings in the operating room included externally rotated and shortened left lower limb. There was one laceration each, on the left leg and right knee. Under general anaesthesia and muscle relaxation, the surgeon carried out “reduction” of left hip dislocation with steinmann’s pin insertion in the left tibia while the lacerations were sutured. On subsequent check x-ray it was discovered that wrong-site surgery had taken place. The patient was reposted for re-manipulation of central fracture/dislocation of the right hip, which was carried out two days after the initial surgery and confirmed by postoperative check x-ray.

### Case 2

A 43 yr old bus driver presented to the trauma unit on November 17, 2012 with inability to bear weight on both lower limbs following road traffic accident 20 hours earlier. Multiple lacerations were seen on the face, scalp and left forearm. There was tenderness over the right wrist, with crepitus. Both lower limbs were externally rotated with swelling of both thighs and left foot. Pelvic contraction-distraction test was negative and urethral catheterization drained clear urine. Radiologic investigation revealed bilateral femoral fracture with left tibial fracture. The Packed cell volume was 19%. He was transfused with two units of blood, and scheduled for open reduction and internal fixation of multiple limb fractures five days post- presentation. The consent for the 1^st^ stage surgery indicated open reduction and internal fixation of fractures of the right femur and tibia. The booking list also indicated the procedure as open reduction and internal fixation of fractures of the right femur and tibia. The Anaesthetist on detecting the incongruency between the diagnosis and the intended procedure had alerted the surgery resident to sort out the concern with his team. Under general anaesthesia, the surgeon on completing the fixation of right femoral intertrochanteric and supracondylar fractures proceeded to make the incision and blunt dissection, for the plating of supposed right tibial fracture. At this point the Anaesthetist notified him of the earlier concern that was raised with the surgery resident. With the displayed x-ray, wrong-site surgery was confirmed and the right leg wound was closed in layers, while open reduction and internal fixation was then carried out on the left tibia with 8-hole broad DCP. A week later, the 2^nd^ stage of the surgery comprising open reduction with 95° condylar plating for the comminuted left femoral supracondylar fracture was carried out under subarachnoid block.

## Discussion

The two index patients had orthopaedic procedures at wrong sites of the body, in the lower extremities. Neither of the incidents was officially reported to the department, nor even mentioned in regular clinical morbidity/mortality review; a reflection of the culture of denial and shame. Consequently no audit or root –cause analysis was instituted. Of the 150 cases of wrong-site surgery reported to JCAHO [6], 126 cases had root cause analysis information of which surgery on the wrong part of the body accounted for 76%, whereas surgery on the wrong patient accounted for 13% and wrong surgical procedure was 11%. Of these by far the largest proportion (41%) relate to orthopaedic surgery. This preponderance of orthopaedic wrong- site surgery is further collaborated by Robinson and Nuir [7]. The remote possibility of a successful legal defence in cases of surgery performed in a wrong limb has been expressed [8] and calls for genuine concern among surgeons in view of the rising awareness in our increasingly litigious society. Whereas wrong- site surgery account for only 2% of all orthopaedic malpractice claims in the USA, as much as 84% of those claims result in a court award to the plaintiff [9]. The surgeon in **Case 1** was a senior resident, while the surgeon in **Case 2** was a Chief Consultant Orthopaedic Surgeon giving credence to the finding by Adeyemo et al. [5] that years of experience of the surgeon was an insignificant factor in wrong- site surgery( P > 0.05). Thus it would not be expedient to relegate wrong- site surgery to an issue of inexperienced surgeons.

The JCAHO had identified risk factors of wrong-site surgery to include emergency cases, physical deformity, multiple surgeons and multiple procedures being performed during a surgical session. Root cause analysis had implicated distraction factors, incomplete preoperative assessment, non –availability of pertinent information in the operating room, policy issues such as surgical site marking and verification checklist in the operating room. Several of these risk factors and root causes were clearly at play in each of the reported cases. System inadequacies were obvious in these cases as the regional trauma centre did not have any institutional protocol in place for surgical patient safety. The JCAHO Universal Protocol for Preventing Wrong- Site, Wrong- Procedure and Wrong- Person surgery recommends three minimum requirements; preoperative verification and site marking in the preoperative holding area, and a “time-out” just before surgery in the operating room. Clarke et al. [10] had found preoperative verification the most effective of the three steps of the Universal Protocol and that the patient was a more reliable source of accurate information than the documents; and that marking the operative site gives the patient a voice even under anaesthesia. The feat of “Operate Through Your Initials” protocol in Canada in reducing wrong-site surgery by 62% in a comparative data from seven years preceding the initiation of the programme in 1994 and seven years post-date [11] lends credence to its effectiveness as a patient-safety tool. Similarly, other studies [12,13] have reported actual decline in morbidity and mortality by about 50%, following the implementation of the “WHO Surgical Safety Checklist”. Treadwell et al. [14] in their study encompassing various Surgical Checklists found that implementation of the strategies was associated with increased detection of potential safety hazards and reduced surgical complications. Whereas Surgical Checklists have been shown to improve safety in the operating room, and adjudged to be simple and desirable by various peri-operative practitioners, sub-optimal implementation remains evident in various settings despite this awareness and enthusiasm [15,16]. The “WHO Surgical Safety Checklist” has since been adopted and implemented as a standard of care in our health facility since 2012 but there is yet no survey or audit to ascertain the level of compliance.

## Conclusion

It is worrisome that as at present, patient safety initiatives such as the JCAHO “Universal Protocol” and the “WHO Surgical Safety Checklist” among others are not being implemented as a standard of care in many parts of Africa, Asia and Europe. In appreciating that wrong-site surgery is more of system lapse than individual failure of the surgeon; the pervading culture of denial, blame and shame in such instances, should necessarily give way to structured systems approach through institutionalisation of patient safety protocols.
